# Personalized Temporomandibular Joint Total Alloplastic Replacement as a Solution to Help Patients with Non-Osteosynthesizable Comminuted Mandibular Head Fractures

**DOI:** 10.3390/jcm13175257

**Published:** 2024-09-05

**Authors:** Paulina Pruszyńska, Marcin Kozakiewicz, Piotr Szymor, Tomasz Wach

**Affiliations:** Department of Maxillofacial Surgery, Medical University of Lodz, 113 Żeromskiego Str., 90-549 Lodz, Poland; marcin.kozakiewicz@umed.lodz.pl (M.K.); piotr.szymor@umed.lodz.pl (P.S.); tomasz.wach@umed.lodz.pl (T.W.)

**Keywords:** fracture diagnosis, personalized fracture treatment, surgical planning tools, mandibular head fracture, TMJ, endoprosthesis, custom-made total alloplastic replacement, CAD/CAM, maxillofacial trauma, temporomandibular joint

## Abstract

**Background**: Treatment methods for mandibular head fractures are controversial, although effective techniques for open reduction and rigid fixation (ORIF) have been known since the late 1990s. Notably, some forms of posttraumatic comminution of the mandibular head can be reduced or fixed. **Methods**: This study presents a personalized treatment to cure patients with nonreduced comminuted fractures of the mandibular head: total temporomandibular joint alloplastic replacement (18 patients). The reference group included patients who underwent ORIF (11 patients). **Results**: Personalized alloplastic joint replacements resulted in a more stable mandibular ramus after three months compared with ORIF. **Conclusions**: The authors recommend not performing osteosynthesis when the height of the mandibular ramus cannot be stably restored or when periosteal elevation from most of the mandibular head is necessary for ORIF. Personalized TMJ replacement should be considered in such patients. Personalized medicine allows patients to maintain a normal mandibular ramus height for a long period of time.

## 1. Introduction

**Figure 1:**
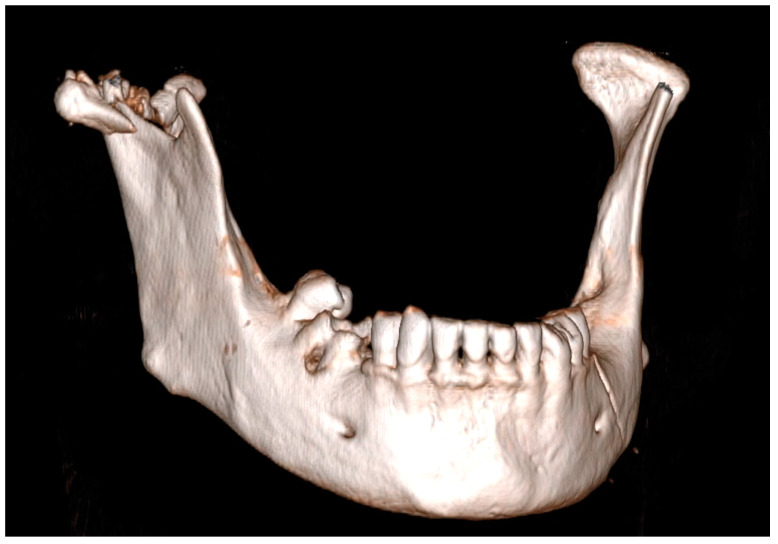
Comminuted mandibular head fracture on right-hand side.

Given the increasingly active lifestyle of the population, facial fractures remain a major aspect of maxillofacial surgery. Based on the newest studies, doctors have much greater possibilities for less invasively treating injuries; however, optimal surgical methods for comminuted fractures have not yet been identified (Figure 1). Comminuted fractures of the mandible are caused by high-impact trauma, which can cause the bone to be broken, splintered or crushed into more than two pieces within the same area of the mandible [1,2].

Considering facial trauma, mandibular fractures occur twice as frequently as fractures of the bones of a midface [3]. Many of these fractures, 21–52%, involve the condyle [4,5,6].

As early as 1971, Korzon and Kruk reported that purely conservative treatment does not always yield good results when evaluating the long-term results of conservative closed treatment for mandibular process fractures. It fails in fractures with significant displacement, full dislocation and especially in old fractures of the mandibular process [7].

Currently, fractures with large displacements, especially dislocations, are not treated with conservative methods [8]. For condylar trauma in general, surgical treatment provides better results than conservative management in the adult population [9,10,11]. The standard strategy to restore condylar process fractures involves the placement of plates in the condylar process or screws in the mandibular head [12]. To date, a variety of fixation devices have been studied, including micro- and miniplates, cannulated screws or bioresorbable pins and screws [13,14,15,16,17,18]. Considering the material used, surgeons often choose between metal and bioresorbable fixation. Classic metal fixations have specific disadvantages. These disadvantages include the potential need to remove these implants, the possibility of causing inflammatory reactions, sensitivity to heat, the possibility of protruding beyond the mandibular head in the course of its physiological remodeling, bone atrophy or screws that are placed too deep. In these cases, capsule damage can also occur. Resorbable materials are an alternative. They have the advantage of not needing to be removed once bone union has occurred, and they can be easily corrected if the screw position is judged to be inadequate. The use of resorbable screws is associated with technical difficulties, e.g., ease of damage to the screw during screwing. Notably, this strategy is plagued by low polymer strength, low fixation stability and inflammatory reactions during the resorption period (sterile abscesses) [19,20,21,22].

Because the accuracy of reduction and fixation is the most important aspect during surgery, finding an effective method to increase precision has been the subject of recent discussions [23].

The treatment of comminuted mandibular head fractures rarely yields ideal anatomical and physiological results because microscopic bone fragments tend to resorb.

The nonanatomical setting of bone fragments causes several disorders in the stomatognathic system. An incorrectly positioned mandibular condylar process causes morphological disorders in the temporomandibular joints, especially on the fractured side, during long-term observation after injury. These fractures irreversibly contribute to the shortening of the mandibular ramus, which is the cause of malocclusion (open anterior, crossbite) [24], bone fragment malunion [25] and/or temporomandibular joint malfunction (limited mouth opening, pain, deviation of the mandible to the affected side when opening) [26].

Mandibular head fractures are among the most difficult fractures to treat [27,28,29]; hence, treatment failure must also be considered by the operating team, which should be prepared for personalized secondary treatment.

In most advanced medical centers, techniques have been developed to individualize treatment and improve the accuracy and quality of fragment reduction and the stability of fixation, with minimal risks of intraoperative complications. Unfortunately, these techniques are not yet popular in many centers. One promising procedure is Pavlychuk’s protocol [30,31]. The authors of this research suggest the use of a titanium patient-specific reinforcement plate, where the first component ensures proper reduction of the condylar head, and the second component is modeled at the lateral surface of the condylar ramus and has a surgical guide for the positioning of two long screws. This research suggests that reinforcement with conventional two-screw fixation of condylar head fractures with a small patient-specific plate acting as a washer may have significant benefits in unfavorable biomechanical conditions.

**Figure 2:**
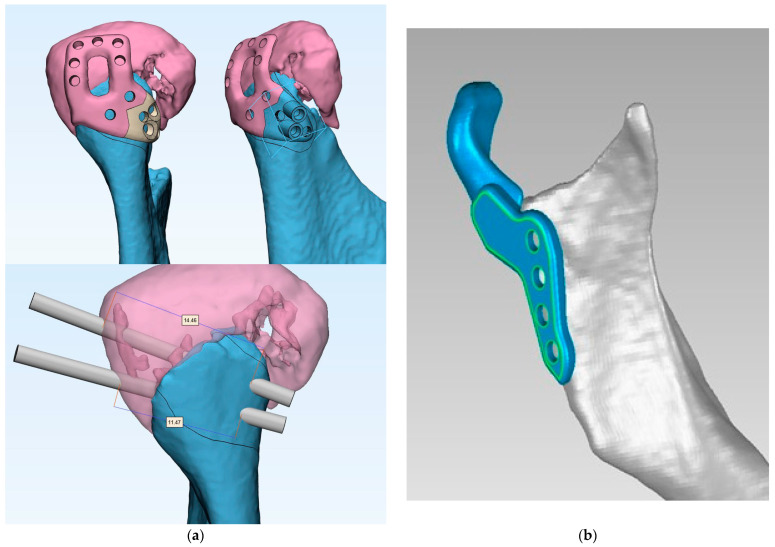
Two options for the management of severe comminuted fractures of the mandibular head: (**a**) Pavlychuk’s protocol [30,31], i.e., CAD/CAM-supported reduction, temporary stabilization and osteosynthesis technique. The fixation method involves the virtual segmentation of bone fragments, application of an individual template for reduction and temporary stabilization; at the same time, the template has guides for inserting positioning screws of pre-planned lengths. Provided courtesy of Tetiana Pavlychuk, DDS, PhD, The Department of Maxillofacial Surgery and Modern Dental Technologies, O.O. Bogomolets National Medical University, Kyiv, Ukraine. (**b**) Example of a temporomandibular joint alloplastic replacement ramus element [32]. In this protocol, all free bone fragments and damaged anatomical structures are removed and replaced with customized temporomandibular joint implants. Figure from the private archive of one of the authors (T.W.).

In such difficult cases, personalized treatment with the use of custom-made temporomandibular joint (TMJ) total alloplastic replacement should be considered as an option (Figure 2).

The aim of this study was to describe a protocol that can be applied in unfixable condylar head fractures and to compare the classic surgical approach with endoprostheses in terms of patients’ functional results. An additional aim was to present a case report that applies the authors’ protocol in a group of patients.

The null hypothesis was that the stability of the height of the mandibular ramus would be the same both after long-screw osteosynthesis and after the use of total alloplastic temporomandibular joint replacement in the treatment of comminuted mandibular head fractures.

## 2. Materials and Methods

### 2.1. Study Group

This retrospective study examined 29 patients with an average age of 38 ± 15 years.

The inclusion criteria were as follows:

- A comminuted mandibular head fracture that could not be treated surgically due to too many miniscule bone particles (cases where significant areas of periosteum would have to be detached in order to reposition the bone fragments and insert screws).
- Failure of previous closed treatment (e.g., temporomandibular joint disorder (TMD) or craniomandibular dysfunction (CMD), osteoarthrosis, pain or ankylosis [29], malocclusion due to mandibular ramus height reduction, lack of stability of previous fixation).

The exclusion criteria were as follows:

- Incomplete medical records;
- Tumors;
- Lack of follow-up (patients who did not present at the second appointment and did not undergo a control CT scan after 3 months);
- Patients suffering from osteometabolic disorders.

All of the patients were divided into two groups:

1. Eighteen patients underwent total TMJ reconstruction with custom-made TMJ implants under general anesthesia. This group consisted of the most recent patients.
2. The reference group consisted of eleven previous patients who were treated with open and internal fixation with mandible head osteosynthesis using long screws according to Kermer [33].

Kermer’s technique was previously utilized at our hospital for severe comminuted head fractures. After the postoperative data (possible loss of mandible ramus height of 4.4 ± 3.3 mm [34]) were collected, the authors recommended a personalized implantation protocol instead. The advantage of such a solution is the precise fit of the implants to the skeleton, without the need to adjust the bones or the implant. Moreover, the locations of screw insertion are planned in advance without colliding with the skull cavity and mandibular canal.

### 2.2. Hospital Procedures

All of the surgeries (in both groups) were conducted by the same operating team with medical antibiotic prophylaxis (amoxicillin or clindamycin in the case of allergy).

Surgical treatment was conducted under general anesthesia with intubation through the nose. The approaches used were the preauricular approach for open reduction internal fixation and the preauricular + periangular mandibular approach for total alloplastic joint replacement. Postoperative monitoring of vital functions (such as pulse oximetry, heart rate and blood pressure measurements) was conducted for the next 24 h.

The postoperative appointments were approximately 7–10 days after the surgery to remove the sutures and 3 months postoperative with control incremental helical computed tomography. Postsurgical physiotherapy continued for 6 weeks to 6 months. All of the patients remained under the care of the outpatient department for one year.

### 2.3. CAD/CAM Procedure for TMJ Implants

**Figure 3:**
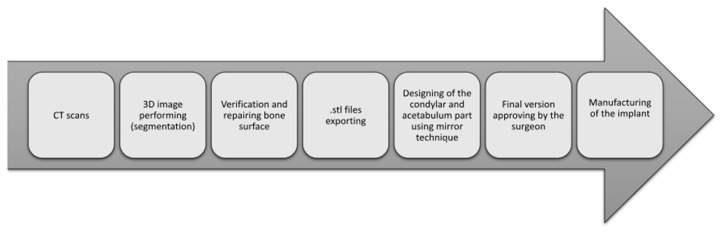
Steps of CAD procedure.

The first step involved performing computed tomography scans (0.6 mm–1 mm; 12-bit DICOM image). The scans included the mandible, maxilla, and temporal bones. Next, the CT scans were used to create a 3D (three-dimensional) image of the hard tissues, a process called segmentation. CAD work was performed using MIMICS (Materialise, Leuven, Belgium). After segmentation, the surface quality of the bone was verified and repaired via the specialized tool Geomagic Studio 14. The 3D bone was then exported as an .stl file, enabling the design of the temporomandibular joint implant design. If a project is unilateral, the mirror technique can be used. In this technique, a mirror image of the healthy condyle from the opposite side is used. Next, the implant fixation part was designed to avoid anatomical structures of the skull and mandible, such as the inferior alveolar nerve and the anterior cranial fossa. Typically, the condylar (ramus) implant requires 8 screws (2.7 mm diameter), whereas the glenoid part is designed with 4 screws (1.5 mm diameter). The length of the screws was individualized for each patient. The inner surface of the condyle was personalized to fit the surface of the mandible ramus. Each step of the design was supervised, and a maxillofacial surgeon was consulted. If the final version of the condylar implant was accepted, it was manufactured via direct metal laser sintering (DMLS) techniques using titanium alloy. The glenoid part of the TMJ was manufactured from UHMW-PE (ultra-high-molecular-weight polyethylene) using CNC milling (computer numerical control). Each step of the CAD procedure is presented in Figure 3.

### 2.4. Evaluation of Treatment

The results of treatment were recorded 3 months postoperation. Radiological evaluation was performed with RadiAnt ver. 2021.1 software (www.radiantviewer.com accessed on 13 July 2024). Changes in the height of the mandibular ramus induced by trauma as well as in the posttraumatic period were measured in millimeters. The maximal incisal opening (MIO) and range of lateral mandibular movements (ispilaterotrusion and contralateralotrusion) were clinically measured. Additionally, occlusion was observed. Facial nerve dysfunction was evaluated via the House–Brackmann scale (1-normal function, 6-total palsy).

Statistical analysis was performed using Statgraphics Centurion 18 (Statgraphics Technologies Inc., The Plains City, VA, USA). One-way analysis of variance was utilized when belonging to one of two groups was established as a factor. Furthermore, comparisons of averages between the immediate postop and 3-month postoperative periods were performed via *t* tests. Because a normal distribution was not detected, medians were compared via the Mann–Whitney U test. The test power was determined, and a *p* value of less than 0.05 was considered statistically significant.

## 3. Results

### 3.1. The Proposed Protocol to Treat Nonfixable Fractures of the Mandibular Head

**Figure 4:**
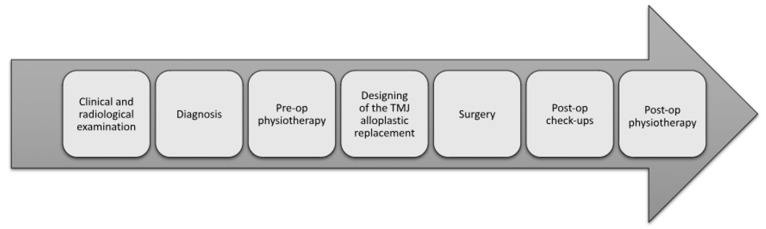
Proposed protocol for treating nonfixable fractures of the mandibular head.

The proposed protocol (Figure 4) to treat nonfixable fractures of the mandibular head includes the following:

1. Clinical and radiological examinations—the clinical diagnosis is always verified via computed tomography (CT) scans.
2. Diagnosis—after the patient’s CT scans and general condition are evaluated, the possible treatment options are discussed. When comminuted mandibular head fractures that cannot be treated surgically are found, the authors begin the endoprosthesis design.
3. Preoperative physiotherapy—taking into consideration that designing and manufacturing takes time, patients undergo preoperative physiotherapy for the short-term maintenance of joint mobility.
4. Surgery—the surgical procedure consists of free bone fragment removal and the implantation of a temporomandibular joint substitute.
5. Postoperative follow-up—all of the patients remain under the care of the outpatient department, where their functional results are being evaluated.
6. Postoperative physiotherapy—patients remain under physiotherapist care to stimulate facial nerve regeneration and improve joint mobility.

### 3.2. An Example of a Clinical Situation

**Figure 5:**
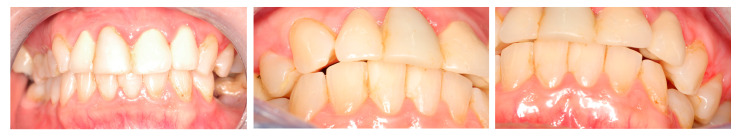
Posttraumatic anterior open bite.

**Figure 6:**
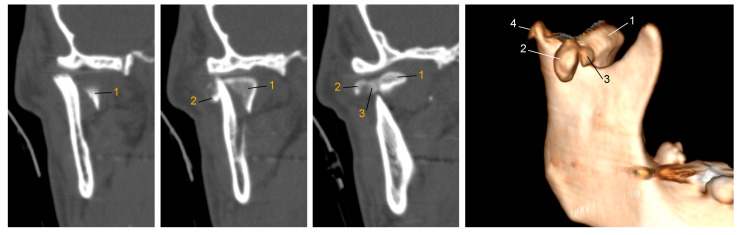
Computer tomography was performed immediately after the injury. Comminuted fracture of the right mandibular head. The bone fragments are numbered from the largest to the smallest.

The following is an example of an approach to treat nonfixable fractures of the mandibular head. After a traffic accident, the patient noted an open bite (Figure 5), and diagnostic imaging showed a comminuted fracture of the right mandibular head (Figure 6).

**Figure 7:**
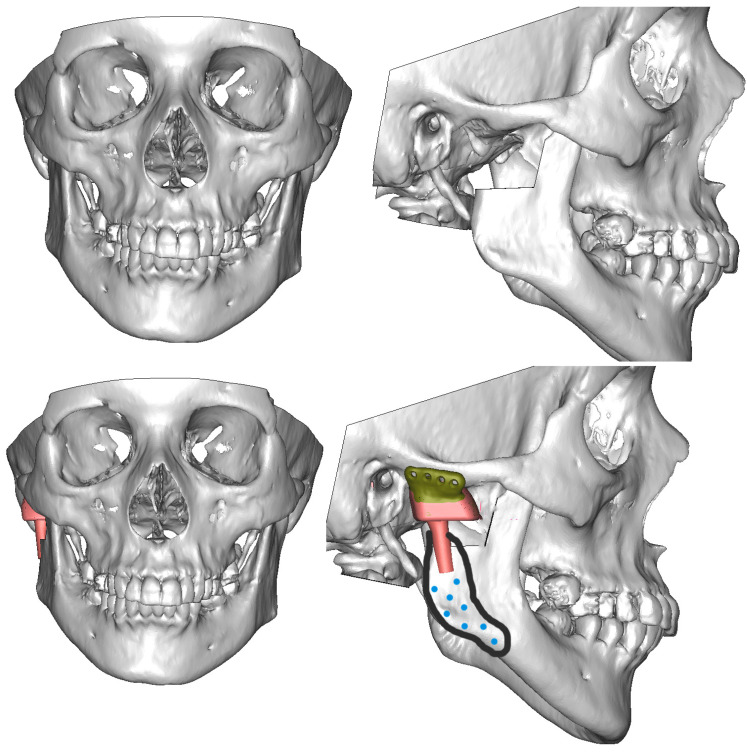
Computer-assisted design of custom-made TMJ implants. During the design phase, the engineer (www.chm.eu accessed on 15 July 2024) communicated with the surgeon, and the design was continuously modified. Blue circles mark the insertion points of the screws that fix the ramus component in such a way as to avoid the perforation of the mandibular canal. After all medical comments were considered, the design was approved and submitted for manufacturing. The ordered individual implants arrived at the hospital within 3–4 weeks.

**Figure 8:**
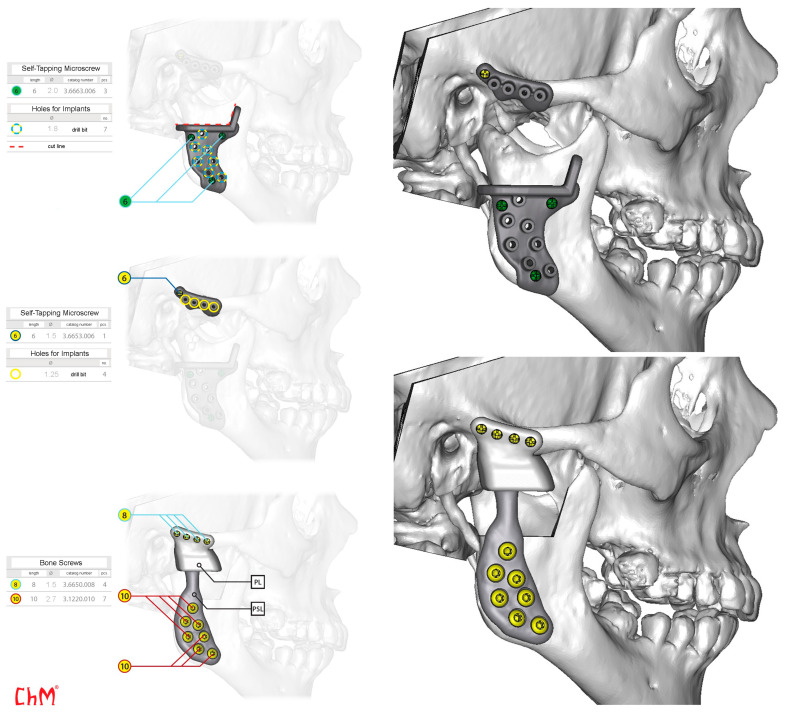
Surgery, implantation and fixation plan. PL—glenoid part; PSL—ramus part, right side.

**Figure 9:**
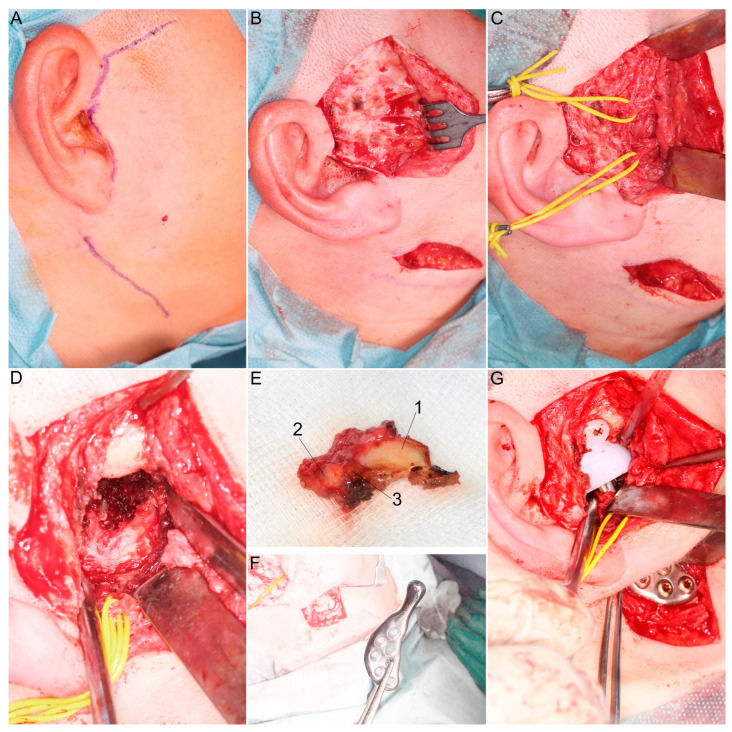
Surgical part of the procedure. (**A**) Two skin accesses are needed: the mandibular periangular for implantation of the ramus part and the preauricular for the glenoid part. (**B**) Skin flap elevation. (**C**) Identification of the facial nerve (here, the zygomatic branch and temporal branch are visible). (**D**) Completion phase of free bone fragment removal, hemostasis and preparation of sites for implants. (**E**) Removed bone fragment described from the largest to the smallest fragment: 1, 2, 3. (**F**) Ramus part prepared for implantation. (**G**) Assembled and fixed alloplastic temporomandibular joint substitute: the glenoid part is made of ultra-high-molecular-weight polyethylene by computer numerical control milling, whereas the ramus part is made with an additive technique from grade 23 titanium alloy.

**Figure 10:**
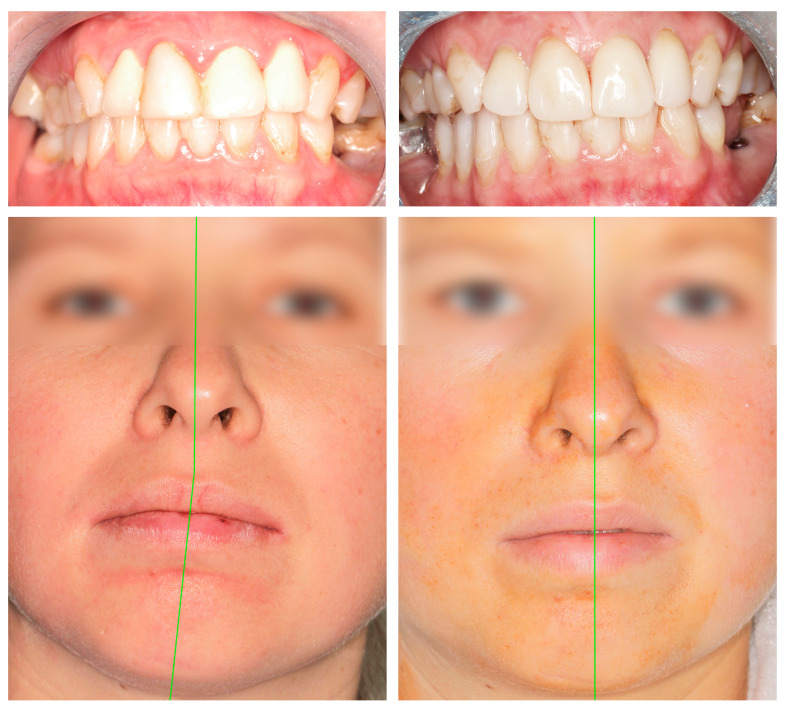
Early clinical results of treatment (preoperative view on the left-hand side and postoperative view on the right-hand side). The face is symmetrical, and occlusion is properly supported. (Permission was obtained to present the patient’s face—it is available from the journal’s editorial office).

After the size and position of the four fragments were evaluated, osteosynthesis was determined to lead to bone resorption within the treated mandibular head. In contrast, closed treatment was not expected to restore the original height of the mandibular ramus. Thus, both classical treatments would have led to dysfunction in the stomatognathic system. In this situation, the treatment of choice is total alloplastic replacement of the temporomandibular joint. We routinely used personalized two-part implants (Figure 7, Figure 8, Figure 9 and Figure 10).

### 3.3. Further Results

This study examined 29 patients, 10 females and 19 males, with an average age of 38 ± 15 years.

Road traffic accidents were the most common reason for comminuted fractures of the mandibular head (followed by falls and interpersonal violence). The average number of proximal mandible head fragments was 1–5 (3.2 ± 1.0) in all 29 patients. All patients presented significant posttraumatic anatomical abnormalities that reflected temporomandibular joint dysfunction (Table 1).

**Figure 11:**
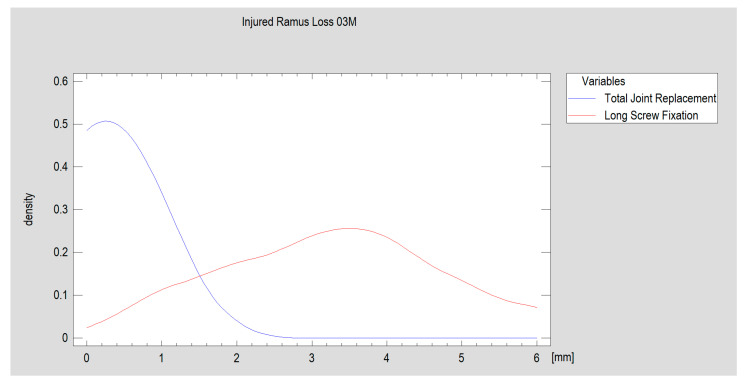
The extent of the early loss of mandibular ramus height. The personalized TMJ solution resulted in less ramus loss (*p* < 0.001, test power 98.7%). Density traces revealed that, in the TJR group, most patients had a measured mandibular ramus height loss of 0.4–0.5 mm 3 months after surgery. In contrast, in the LSF group, most patients experienced a ramus height loss of as much as 3–4 mm.

**Figure 12:**
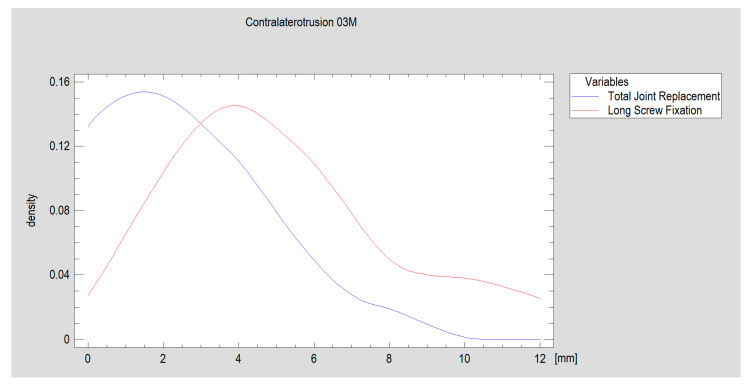
The size of the early recovery of mandibular movement on the side opposite the injured side. A personalized TMJ solution was associated with a worse functional result (*p* < 0.05, test power 38.1%). The density traces revealed that, in the TJR group, most patients had a measured movement of 1–1.5 mm laterally, whereas in the LSF group, lateral movements of approximately 4 mm were observed.

Selected features of the structure and function of the temporomandibular joint region were evaluated 3 months after surgery. Variables such as ramus shortening, maximal interincisal opening, ipsilaterotrusion, contralaterotrusion and facial nerve dysfunction were compared. The results, divided into a group treated with personalized joint implants and a reference group treated with long screw fixation, are shown below (Table 2 and Figure 11 and Figure 12). The fragmentation of the heads (average of three proximal loose fragments each, *p* = 0.91) and the frequency of bilateral fractures (in the personalized treatment group: four and in the open reduction internal fixation-treated group: five; *p* = 0.94) were equal between groups. Three months after treatment, most patients had an occlusion as that before the injury. In the personalized treatment group, two crossbites were observed, and in the ORIF-treated group, two open bites were observed (the results remained at the threshold of statistical significance, i.e., *p* = 0.06).

Thus, significantly better results were observed with personalized treatment (Figure 10), but lateral movement of the mandible did not significantly return to the opposite side of the injury in these patients. A significantly greater degree of laterotrusion was observed in the group of patients who were fixated with long headless screws than in the total alloplastic temporomandibular joint replacement group (Figure 11).

## 4. Discussion

To date, an ideal solution for managing condylar head fractures has not been identified [35]. Considering the available options, surgical treatment is inarguably superior to conservative treatment for moderately displaced condylar fractures [36].

The goal of surgical treatment is to eliminate anatomical disturbances, which should result in functional recovery [37]. Anatomical purposes include the following corrections:

1. a decrease in the height of the mandibular ramus,
2. the loss of joint surface contact between the condylar head and the glenoid fossa and
3. the dislocation of the articular disc in the medial–anterior direction.

These three anatomical defects cause functional deterioration: mastication due to bite changes in the teeth (permanent crossbite on the trauma side or open anterior bite), intra-articular hematoma together with the risk of ankylosis (full and permanent immobilization of the joint).

Although surgical treatment of condylar head fractures is not a new procedure in maxillofacial surgery, various strategies to manage these fractures remain a topic of discussion [38], and several different approaches have been described to this end [39]. The two most commonly used surgical approaches are the preauricular [40] and retroauricular approaches [41]. The former provides excellent visibility and access to bone fragments; however, its main risk is the possibility of facial nerve injury. The retroauricular approach is an option for bypassing the facial nerve and is also a quick procedure; however, it leaves the possibility of auriculotemporal nerve paralysis and auricular canal stenosis.

Research has also been conducted to evaluate the quality of bone union when various materials are used for fixation. Magnesium screws yield results similar to those of traditional titanium fixation in terms of bone union quality [42]. Nevertheless, other authors reported that titanium alloys cause dysfunction and redox imbalance in the mitochondria of fibroblasts [43]. In addition, polymer fixations of the mandibular head have been popular for years [44]. Thus, new materials need to be identified. Currently, attempts are being made to create screws from bone tissue to fix fractures [45,46], which is promising for future surgeons.

There are no methods for closed reduction of comminuted mandibular head fractures to repair the condition of the stomatognathic system; hence, surgical procedures are chosen. Attempting open fixation of the fine-spaced mandibular head may sometimes lead to the formation of an open bite within 3 months after surgery due to slow bone loss from the mandibular head [47]. Therefore, comminuted fractures of the mandibular head cause an irreversible decrease in the height of the mandibular ramus [48], which causes significant functional disorders of the stomatognathic system. The most important of these disorders are open anterior bite, pain and joint ankylosis. These disorders are not a risk with alloplastic arthroplasties, but here, the contralateral dentition is lost. Both attempts to fix comminuted fractures with long positioning screws and alloplastic joint replacements carry some risk of permanent facial nerve dysfunction (approx. 1% [39]). In the former, this dysfunction is related to the great difficulty in finding, reducing and rigidly fixing all fragments of the mandibular head. In the latter, the dysfunction is related to the extent of the surgical procedure.

In the epidemiology of ankylosis, mandibular head fractures account for 26% of cases of this pathology in children and 77% of cases in adults [40]. Moreover, even dislocation of the disc leads to restricted mobility in the temporomandibular joint by another mechanism, i.e., the limitation of the interincisal opening to 25 mm. This restriction is due to a loss of contact disk-to-glenoid fossa surface and mandible movement toward the healthy side because of the severe dislocation of the distal insertion of the pterygoid muscle [24,25,26,49]. Therefore, ramus height restoration and articular surface improvement, together with intra-articular disk reduction, lead to good functional treatment results.

Unfortunately, clinically achieving the aforementioned anatomical goals is often impossible for comminuted fractures of the mandibular head. The Kermer [33] protocol should be applied to simpler fractures of the mandibular head, not multifracture with many small fragments or crush fractures. Despite heroic and unpromising long screw fixation, two computer-assisted options remain as options:

1. Pavlychuk’s protocol [30,31];
2. Custom-made alloplastic total TMJ replacement [32].

To date, long-term results of the Ukrainian protocol have not been presented because of the war. The technique is interesting and promising, and surgeons await further outcome reports. Pavlychuk’s protocol saves much more tissue than the total joint replacement procedure and offers a chance to avoid the need for endoprostheses.

Total alloplastic TMJ replacement is a well-known procedure that has already been evaluated by researchers. A retrospective study [50] that assessed pain, diet, function and quality of life (QoL) confirmed that in most patients, diet consistency scores and QoL improved and pain scores decreased. Moreover, an analysis of objective data in the cited study demonstrated improvements in mandibular motion.

The use of endoprostheses appears to provide a nonresorbable mandibular frame that maintains the vertical dimension of the occlusion. The risks associated with surgery are lowest when only the primary procedure is performed: there is no scar, the vascularization of the tissue is preserved and there is no secondary contracture of the lateral pterygoid muscle [26].

The disadvantages of reapproaching the mandibular head after failed open reduction and internal fixation (ORIF) are as follows: scars around the facial nerve, displaced screws, bone-drenched screws, damaging the skull base with fixing material, skin fistulas, displacement of the teeth by muscular force, tooth damage [51] and prosthetic restorations that need to be changed after restoration of the ramus height. For these reasons, performing a single and definitive operation is preferred.

Every surgical intervention is known to carry the possibility of short-term and long-term complications. Postoperative complications associated with total TMJ replacement include infection, allergy to metal, loosening of screws or implants, implant fracture or reankylosis of surrounding hard tissue [52]. Currently, the most commonly used alloys for TMJ replacement are Co-Cr-Mo or titanium grade 23. Studies have examined differences in the early outcome of treatment when all titanium prostheses (in patients allergic to metal) and Co-Cr-Mo prostheses (in patients not allergic to metal) are used; outcomes are similarly favorable [53]. However, some studies have reported that the foreign body response to the metal used in TMJ implants can be observed in the long term [54,55].

With respect to the negative administrative side of this treatment process, the high cost and availability of software tools need to be considered [56]. In Poland, individual approval of the National Health Fund is needed to finance such a procedure. But on the other hand, comminuted fractures significantly increase surgery duration. Prolonged operation times should be avoided due to the associated complications and negative effects on the efficiency of the use of operating room resources [57].

Considering all of these side effects, surgeons can currently minimize the risk of medical complications with personalized treatment. With the use of computed tomography and accurate manufacturing of implants via CAD/CAM solutions, customized implants that can be perfectly fixed to patients’ bones [58] and simultaneously meet the surgeon’s requirements can be produced. Individualization limits postoperative and long-term side effects while restoring satisfactory function.

With respect to the future of maxillofacial surgery, efforts have been made to produce temporomandibular joint replacement via the use of bioengineered tissues. Due to advancements in this field (like biomimic surfaces), viable bioengineered TMJ components can be reproduced [59].

Unquestionably, the limitation of the study is that the presented results are early results of the treatment. Moreover, this sample contains only 29 patients, which can be considered a small group. However, even from such a small group, the authors could show with great certainty (*p* < 0.001, test power 98.7%) that the results regarding low ramus shortening are very promising in non-fixable fractures treated by TMJ replacement. For full statistical inference, a significantly larger sample will need to be studied. Lastly, the study is also a single-center study.

## 5. Conclusions

The authors recommend not performing osteosynthesis when the height of the mandibular ramus cannot be stably restored or when periosteal elevation from most of the mandibular head is necessary for ORIF. Personalized TMJ replacement should be considered in such patients. Personalized medicine allows patients to maintain normal mandibular ramus height and stable occlusion for a long period of time. An ideal method to treat comminuted mandibular head fractures has not yet been established because some aspects of stomatognathic function, e.g., contralateral motion, do not return to normal after such an injury, even with custom-made TMJ replacement surgery.

## Figures and Tables

**Table 1:** General information of all presented patients.

Parameter	Before Treatment	Immediately Postop	3 Months Postop
Ramus Shortening [by mm]	11 ± 11	0.0 ± 0.0	1.5 ± 1.8
Maximal Interincisal Opening [mm]	23 ± 13	19 ± 8	29 ± 7
Ipsilaterotrusion [mm]	4.5 ± 5.5	4.0 ± 3.2	5.3 ± 7.1
Contralaterotrusion [mm]	1.1 ± 2.3	2.5 ± 1.7	3.5 ± 2.9

**Table 2:** Results of treatment with personalized total TMJ replacements versus a reference group, i.e., patients in whom fixation of multifractures with long screws was attempted.

Parameter	Group	Before Treatment	Immediately Post-Op	3 Months Post-Op	00 M-03 M Improvement
Ramus Shortening [by mm]	Total Joint Replacement	15.1 ± 13.5	0.0 ± 0.0	0.3 ± 0.4 ^#^	*p* < 0.05
	Long Screw Fixation	7.5 ± 5.4	0.0 ± 0.0	3.3 ± 1.5 ^#^	*p* < 0.05
Maximal Interincisal Opening [mm]	Total Joint Replacement	23.8 ± 15.9	17.1 ± 6.6	27.3. ± 7.8	*p* < 0.05
	Long Screw Fixation	22.1 ± 6.7	20.3 ± 8.8	31.9 ± 5.3	*p* < 0.05
Ipsilaterotrusion [mm]	Total Joint Replacement	5.3 ± 5.4	3.7 ± 3.3	5.3 ± 4.2	n.s.
	Long Screw Fixation	3.0 ± 5.9	4.3 ± 3.3	5.2 ± 3.0	n.s.
Contralaterotrusion [mm]	Total Joint Replacement	1.3 ± 2.6	1.7 ± 1.6 ^#^	2.3 ± 2.2 ^#^	n.s.
	Long Screw Fixation	0.9 ± 1.5	3.3 ± 1.6 ^#^	5.1 ± 2.9 ^#^	n.s.
Facial Nerve Disfunction	Total Joint Replacement	1.0 ± 0.0	3.5 ± 0.7	1.9 ± 0.9 ^#^	*p* < 0.05
	Long Screw Fixation	1.0 ± 0.0	2.9 ± 0.8	1.3 ± 0.6 ^#^	*p* < 0.05

#—Statistically significant difference between groups. 00 M—immediately postoperation. 03 M—three months postoperation. n.s.—lack of statistical significance.

## Data Availability

The data on which this study is based will be made available upon request at https://www.researchgate.net/profile/Marcin-Kozakiewicz (accessed on 13 July 2024).
